# The Analysis of ECE1 and PPARG Variants in the Development of Osteopenia and Osteoporosis in Postmenopausal Women

**DOI:** 10.3390/biomedicines12071440

**Published:** 2024-06-27

**Authors:** Izabela Uzar, Anna Bogacz, Małgorzata Łuszczyńska, Marlena Wolek, Katarzyna Kotrych, Andrzej Modrzejewski, Bogusław Czerny, Paweł Ziętek, Adam Kamiński

**Affiliations:** 1 Department of General Pharmacology and Pharmacoeconomics, Pomeranian Medical University in Szczecin, Żołnierska 48, 71-230 Szczecin, Poland; uzari@wp.pl (I.U.); bczerny@wp.pl (B.C.); 2 Department of Pharmacology and Toxicology, Institute of Health Sciences, Collegium Medicum, University of Zielona Góra, Zyty 28, 65-048 Zielona Góra, Poland; 3 Department of Personalized Medicine and Cell Therapy, Regional Blood Center, Marcelinska 44, 60-354 Poznan, Poland; 4 Department of Stem Cells and Regenerative Medicine, Institute of Natural Fibres and Medicinal Plants, Kolejowa 2, 62-064 Plewiska, Poland; mg.luszczynska@gmail.com (M.Ł.); marlena.wolek@iwnirz.pl (M.W.); 5 Department of General and Dental Radiology, Pomeranian Medical University in Szczecin, Powstańców Wielkopolskich 72, 70-111 Szczecin, Poland; kotrych1@gmail.com; 6 Clinical Department of General Surgery, Pomeranian Medical University in Szczecin, Piotra Skargi 9−11, 70-965 Szczecin, Poland; amodrzejewski@interia.pl; 7 Department of Orthopaedics, Traumatology and Orthopaedic Oncology, Pomeranian Medical University in Szczecin, Unii Lubelskiej 1, 71-252 Szczecin, Poland; pawelziet@gmail.com; 8 Department of Orthopedics and Traumatology, Independent Public Clinical Hospital No. 1, Pomeranian Medical University in Szczecin, Unii Lubelskiej 1, 71-252 Szczecin, Poland; emluc@wp.pl

**Keywords:** osteoporosis, allelic variants, *PPARG*, *ECE1*, polymerase chain reaction

## Abstract

Osteoporosis is a multifactorial systemic skeletal disease that is characterized by a low bone mineral density (BMD) and the microarchitectural deterioration of bone tissue, leading to bone fragility. The search for new genes that may play an important role in the regulation of bone mass and the development of osteoporosis is ongoing. Recently, it was found that altering the activity of the endothelin-1-converting enzyme encoded by the *ECE1* gene may affect bone mineral density (BMD). Another gene involved in the process of osteoblast differentiation and maturation is believed to be *PPARG* (peroxisome proliferator-activated receptor gamma). This participates in regulating the transformation of stem cells and affects the process of bone formation and resorption. Therefore, we analyzed the association of the *ECE1* and *PPARG* variants with osteopenia and osteoporosis risk in the Polish population. This study included a group (*n* = 608) of unrelated Polish women (245 individuals with osteoporosis (aged: 57 ± 9), 109 individuals with osteopenia (aged: 53 ± 8) and 254 healthy controls (aged: 54 ± 8)). The real-time PCR technique was used to determine the genetic variants for rs213045 (-338*G*>*T*) and rs213046 (-839*A*>*C*) of the *ECE1* gene and rs1801282 (Pro12Ala, *C*>*G*) of the *PPARG* gene. Analysis of the *PPARG* rs1801282 variants did not show any association with the risk of osteoporosis and osteopenia. However, in the densitometric results, lower median Z-score values were observed for the *T* allele compared to the *G* allele for the rs213045 variant of the *ECE1* gene (−1.11 ± 1.07 vs. −0.78 ± 1.21, *p* = 0.021). Moreover, the *TT* genotype for the rs213045 variant was more common in women with osteopenia (13.8%, OR = 2.82, *p* < 0.05) and osteoporosis (7.8%, OR = 1.38, *p* > 0.05) compared to the control group (5.5%). Additionally, our results suggested that the T allele of rs213045 was more common in women with osteopenia compared to the controls. We further observed that the haplotype containing two major *GA* alleles of *ECE1* (rs213045, rs213046) could reduce the risk of osteopenia in our population. Finally, we found that women with osteoporosis had statistically significantly lower body mass and BMI values compared to the control group. Our results suggest that the *ECE1* rs213045 variant may increase the risk of osteopenia. However, the data obtained require confirmation in further studies.

## 1. Introduction

Osteoporosis is a multifactorial systemic skeletal disease characterized by a low bone mineral density (BMD) and microarchitectural deterioration of bone tissue, leading to bone fragility [1]. The pathomechanism of osteoporosis is complex and multifactorial, associated with changes in osteoclasts’ resorption hyperactivity in relation to the bone-forming cells [2]. 

The bone geometry, microarchitecture, and size are factors that influence the ability of bones to withstand injury. However, 75–90% of the variance in bone strength is related to bone mineral density [3]. As BMD decreases with age, primary osteoporosis occurs mainly in postmenopausal women and older men between the ages of 75 and 80 years [1].

There are hundreds of genetic variants associated with BMD or osteoporosis, which have been identified in genome-wide association studies (GWASs), e.g., *WNT16*, *EN1*, *PKD2L1*, and *JAZF1* [4]. The main genes associated with a higher risk of osteoporosis are *VDR*, *ESR1*, *ESR2*, *COL1A1*, *BMP2*, *TLR*, *STAT1*, and *LRP5* [5,6,7,8,9]. The search for new genes that may play an important role in the regulation of bone mass, and the development of those for osteoporosis is ongoing. 

Recently, it was found that altering the activity of the endothelin-converting enzyme 1 encoded by the *ECE1* gene may have an effect on bone mineral density (BMD) [10]. Endothelin-converting enzyme 1 is a membrane-bound metalloprotease that can cleave a biologically inactive precursor to endothelin-1 (END-1) to form active END-1 [10,11]. Endothelins (ENDs) are peptides with very strong vasoconstrictor properties. However, by stimulating the production of nitric oxide, they may indirectly have a vasodilating effect [11,12,13]. ENDs increase the secretion of aldosterone, adrenocorticotropin (ACTH), and female sex hormones, and they also modulate the inflammatory response by activating neutrophils and increasing the production of pro-inflammatory cytokines. Additionally, END-1 has been shown to lead to an increase in RANKL production in osteoblasts through osteoclastogenesis [13]. In previous in vitro, ex vivo, and in vivo studies, it has already been demonstrated that END-1 signaling influences bone physiology.

Furthermore, a GWAS revealed that the *WNT4* rs7521902 variant located near the *ECE1* gene is significantly associated with the bone mineral density of the lumbar spine and femoral neck, as well as fractures in women [4,10]. The gene involved in osteoblast differentiation and maturation is believed to be *PPARG* encoding peroxisome proliferator-activated receptors gamma, which inhibits their differentiation and stimulates adipocyte maturation. *PPARG* plays an important role in maintaining bone mass. It participates in the regulation of the transformation of stem cells and affects the process of bone formation and resorption [14]. *PPARG* is a transcription factor associated with the metabolism of glucose, lipids, and inflammation and controls the differentiation of osteoblasts and osteoclasts. Increased *PPARG* activity results in bone loss and even a double increase in the risk of fractures in women with diabetes. The mechanism of the adverse impact of *PPARG* on bones is not entirely clear. However, it is known that active *PPARG* increases the resorptive activity of osteoclasts [15]. There is also a connection between *PPARG* and the expression of the sclerostin gene in osteocytes [16]. Sclerostin produced in osteocytes regulates bone formation and resorption by inhibiting WNT signaling and RANKL production. *PPARG*, as a transcription factor regulating sclerostin production, may become a therapeutic target in osteoporosis therapy [15]. Moreover, another study suggested that the *PPARG* rs12497191, rs1801282, and rs3856806 variants may be associated with osteoporosis, especially the common rs1801282 variant [17]. 

Due to the possible influence of the *ECE1* gene on bone mineral density and the *PPARG* gene on the maintenance of bone mass, we selected several variants of these to assess their influence on the development of osteoporosis. The aim of this study was to analyze the effect of the *ECE1* and *PPARG* variants on bone mineral density in postmenopausal women.

## 2. Materials and Methods

### 2.1. Patients

**Figure 1:**
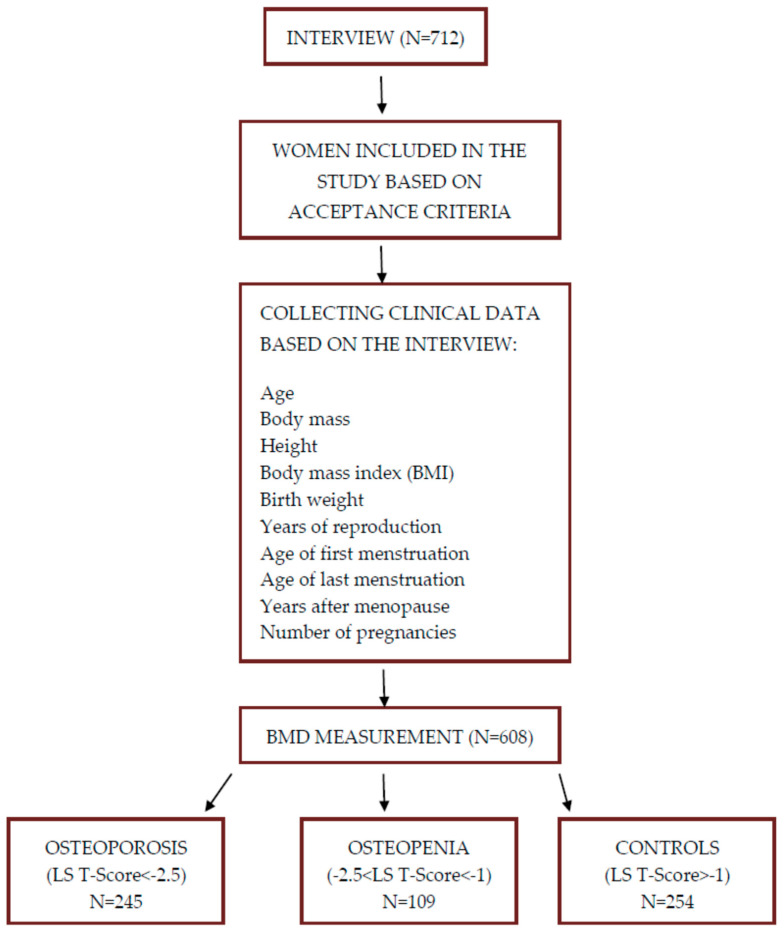
Scheme of clinical data collection.

This study included a group (*n* = 608) of unrelated Polish women (245 individuals with osteoporosis (aged: 57 ± 8), 109 individuals with osteopenia (aged 53 ± 8), and 254 healthy controls (aged 54 ± 8)), who reported to Clinical Hospital No. 1 at the Pomeranian Medical University in Szczecin. Patients were recruited between 2013 and 2015. A detailed interview was conducted with each patient to obtain information on the presence of the disease, use of medications, age of the first and last menstruation, number of pregnancies, birth weight, and smoking. Additionally, weight and height were measured to calculate the body mass index (BMI) according to the appropriate formula (body weight/height^2^) (Figure 1). 

The analysis included all the patients who had reached the menopause at least one year previously, who were not undergoing therapy affecting bone mass, including drugs such as selective estrogen receptor modulators (SERMs), calcitonin, biphosphates, heparin, steroids, thyroid hormones, antiepileptic drugs, GnRH analogues, tibolone, anti-resorptive drugs, statins, and ACE inhibitors, and who had not undergone hormone replacement therapy (HRT). This study excluded patients with abilateral ovariectomy, as well as women suffering from endocrine and metabolic disorders, hematologic diseases, neoplastic disease, kidney diseases, autoimmune diseases, or connective tissue diseases, due to the possibility of these conditions affecting bone mass loss. 

Written informed consent was obtained from all participants. This study was approved by the Bioethics Committee of the Pomeranian Medical University in Szczecin (No. 1415/03 (158/06)). This study was in line with the Declaration of Helsinki (2013).

### 2.2. Determination of Bone Mineral Density

The women were subjected to densitometric tests in the Densitometry Laboratory at Clinical Hospital No. 1 at the Pomeranian Medical University in Szczecin. Bone mineral density (BMD) was determined in the lumbar spine from L2 to L4 using the Dual Energy X-ray Absorptiometry (DEXA) method. Densitometry studies were performed using a LUNAR DPX 100 apparatus (Lunar Corp., Madison, WI, USA). The results of the BMD measurements were expressed in g/cm^2^ and presented by means of LS T-scores and LS Z-scores, which refer to mean values for BMD in a given age group. The bone mineral density measurement value using the DEXA method was assumed to be correct, being between 1 standard deviation from the age mean in relation to the peak bone mass (−1 < LS T-score > +1). On the basis of these measurements, women were classified into the following groups: osteopenia (−2.5 < LS T-score < −1), osteoporosis (LS T-score< −2.5) and normal LS T-score—control (LS T-score> −1). Ratios of the mean BMD to the mean for young adults (YA) and by age (age-matched, AM) were also assessed. 

### 2.3. Genetic Analysis of ECE1 and PPARG Genes

In our study, the real-time PCR technique was used to determine the rs213045 (-338*G>T*) and rs213046 (-839*A>C*) variants of the *ECE1* gene and the rs1801282 (Pro12Ala, *C>G*) variant in the *PPARG* gene. Blood samples were collected in EDTA-containing tubes at the Department of Orthopedics and Traumatology at the Pomeranian Medical University in Szczecin. The analysis of the *ECE1* and *PPARG* variants was carried out at the Department of Stem Cells and Regenerative Medicine, Institute of Natural Fibers and Medicinal Plants in Poznań. Genomic DNA was isolated from peripheral blood using the QIAamp Blood Kit (Qiagen GmbH, Hilden, Germany). DNA concentration was measured with a DeNovix DS-11 spectrophotometer (DeNovix Inc., Wilmington, DE, USA). The LightCycler FastStart DNA Master HybProbe test (Roche Diagnostics, Filderstadt, Germany) and the LightCycler^®^480 device for genotyping the *ECE1* and *PPARG* genes were used. The *ECE1* rs213045 (-338*G>T*) and rs213046 (-839*A>C*) variants and the *PPARG* rs1801282 (Pro12Ala, *C>G*) variant were determined using LightSNiP (TIBMolbiol, Berlin, Germany). PCR was performed in a volume of 10 µL of the reaction mixture according to the manufacturer’s protocol. The conditions were initial denaturation at 95 °C for 10 min and 35 cycles as follows: denaturation at 95 °C for 10 s, hybridization at 60 °C for 10 s, extension for 15 s at 72 °C, and melting for 30 s at 95 °C and 40 °C for 120 s. Allelic variants of the *ECE1* and *PPARG* genes were observed as different melting curves of the PCR products using LightCycler480 software version 1.5.

### 2.4. Statistical Analysis

Statistical analysis was performed using SPSS Statistics 17.0 for Windows. For quantitative variables, an analysis of conformity to the Gaussian distribution was carried out using the Shapiro–Wilk normality test and presented as the mean ± SEM. In the case of conformity of the trait distribution to the normal distribution, a one-way analysis of variance for unrelated variables (ANOVA) was used to assess the relationship between the means in the study groups, followed by the Tukey HSD post hoc test. Categorical variables were presented as numbers (percentages) and were compared according to the abundance of expected values using Pearson’s χ^2^ test. All statistical tests performed were two-sided. Values of two-sided *p* < 0.05 were considered statistically significant. Pearson’s χ^2^ test was used to check whether each of the studied genetic variants met the assumptions of the Hardy–Weinberg equilibrium (HWE). The associations of the studied SNPs with the risk of osteopenia and osteoporosis were assessed using unconditional logistic regression analysis and presented as odds ratios (ORs).

## 3. Results

### 3.1. Clinical Characteristics

A total of 608 participants were included in this study, 254 women with a normal bone mass (controls), 109 with osteopenia, and 245 osteoporosis. The characteristics of the women included in this study are given in Table 1. Patients with osteoporosis were older (*p* = 0.007) and their average birth weight was lower (*p* = 0.001) than the other study participants. We found no differences between the groups in the occurrence of the first menstrual period, but the last menstrual period was the earliest in women with osteoporosis (*p* = 0.037). The participants had a similar number of pregnancies in their lives (median 2, *p* = 0.941). Statistically significant differences between the groups were observed when analyzing growth, weight, and BMI and comparing bone densitometry results. 

### 3.2. Association of PPARG and ECE1 Gene Variants with Susceptibility to Osteoporosis

The frequencies of genetic variants analyzed in this study were consistent with the Hardy–Weinberg equilibrium and comparable to other European populations. The results are presented in Table 2. 

The allele frequencies of the rs1801282 (*PPARG*), rs213045, and rs213046 (*ECE1*) variants in osteopenia, osteoporosis, and controls are presented in Table 3. A statistically significant difference was observed for the rs213045 (*ECE1*) variant. The *T* allele was more common in women with osteopenia compared to the controls (0.317 vs. 0.242, crude OR = 1.45, 95%CI: 1.02–2.06, *p* = 0.037).

The *GG*, *GT*, and *TT* genotypes of the *ECE*1 rs213045 variant were 57.1%, 37.4%, and 5.5% in the controls and 50.5%, 35.8%, and 13.8% in women with osteopenia (*p* = 0.037). With the *GG* genotype as a reference, the codominant and recessive crude model were associated with an increased risk of osteopenia (*p* < 0.05). After adjustment according to patients’ ages and BMIs, there was no statistically significant difference between the osteopenia group and controls (Table 4).

**Figure 2:**
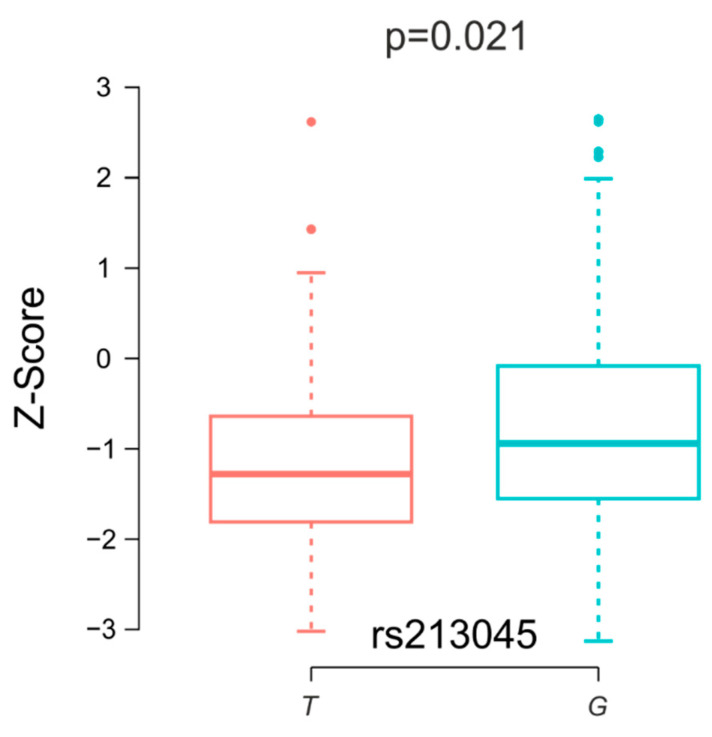
The box plot of Z-score median values observed for the alleles *ECE1* rs213045.

When comparing clinical and densitometric parameters depending on the patients’ genotypes, no statistically significant differences were found. The analysis of these parameters depending on the allele distribution indicated a statistically significantly higher BMI in carriers of the *G* allele of the rs1801282 variant (25.58 ± 3.90 vs. 24.65 ± 3.94 kg/m^2^ for *C* allele, *p* = 0.040). Interestingly, in both polymorphic variants of the *ECE1* gene, minor alleles were associated with lower birth weights of the study participants. For allele *T* (rs213045), the birth weight was 3154.25 ± 549.48 g, and for *G,* it was 3399.19 ± 470.23 g (*p* = 0.011), whereas for *C* (rs213046), it was 3019.09 ± 434.11 g, and we found a result of 3350.35 ± 506.30 g for allele *A* (*p* = 0.038). The only statistically significant differences observed in the densitometric results were lower median Z-score values for the *T* allele compared to the *G* allele for the rs213045 variant of the *ECE1* gene (−1.11 ± 1.07 vs. −0.78 ± 1.21, *p* = 0.021) (Figure 2).

#### Analysis of Linkage Disequilibrium and Haplotype Frequencies

**Figure 3:**
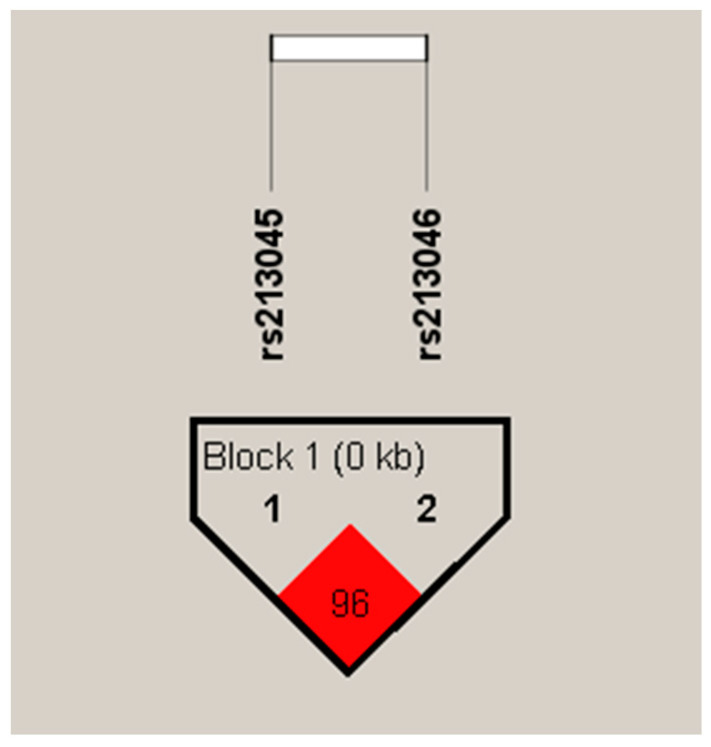
Linkage disequilibrium plot between *ECE1* gene variants generated by Haploview software. Linkage disequilibrium (LD) is displayed as pairwise D’ values.

The linkage disequilibrium and haplotype frequencies of the *ECE1* gene were analyzed using HaploView 4.2 software (http://www.broad.mit.edu/mpg/haploview/, accessed on 22 February 2023). The *ECE1* gene variants analyzed in this study are separated by a distance of 501 base pairs and are in linkage disequilibrium (D’ = 0.96, LOD = 37.2, r^2^ = 0.22) (Figure 3). 

The haplotype frequencies of *ECE1* gene variants’ different comparison groups are summarized in Table 5. The results showed that the haplotype containing two major *GA* alleles (rs213045, rs213046) reduces the risk of osteopenia in our population (OR = 0.68, 95%CI: 0.48–0.97, *p* = 0.032). 

## 4. Discussion

Osteoporosis is a disease associated with both a low bone mass and an increased risk of fractures. Although much research is underway, little is known about the influence of genetic factors on bone growth and formation. In order to learn more about the molecular mechanisms, polymorphic variants of genes that may increase the risk of osteoporosis are still being sought [18]. In our study, we analyzed the influence of the *ECE1* rs213045 and rs213046 variants and the *PPARG* rs1801282 variant on the occurrence of osteopenia and osteoporosis in postmenopausal women. Patients with osteoporosis were older (*p* = 0.007) and had a lower birth weight (*p* = 0.001), and their last menstruation occurred earlier compared to the control group (*p* = 0.037).

In the case of BMI values, the analysis showed statistically significant differences between the studied groups (osteoporosis: 23.80 ± 3.09, osteopenia: 24.67 ± 3.97 vs. the control group: 26.04 ± 4.55, *p* < 0.05), indicating that women with osteoporosis had lower BMI values compared to healthy women. Woman with osteoporosis had statistically significantly lower weights compared to the control group (*p* < 0.05). Similarly, Hansen et al. observed statistically significant differences related to body weight, which was the lowest in women with osteoporosis, and age, which was the highest in women with osteoporosis in comparison to the control group and patients with osteopenia [10].

Analysis of the *PPARG* rs1801282 variants revealed no relationship between the controls and the study group (osteopenia and osteoporosis). The analysis of clinical and densitometric parameters depending on the allele distribution indicated a statistically significantly higher BMI in carriers of the *G* allele of the *PPARG* rs1801282 variant (*p* = 0.040). Similarly, in a study of metabolic biomarkers in patients with type 2 diabetes, Reza-Lopez et al. described that the *G* allele is associated with a higher BMI and greater waist circumference than in *CC* homozygotes. It is possible that the rs1801282 *PPARG* polymorphism modulates adiponectin expression. However, results vary according to the population, ethnicity, individual characteristics, and other conditions [19]. Li et al., in a meta-analysis on the significance of *PPARG* polymorphisms in obesity and hypercholesterolemia, also showed that carriers of the *G* allele of the rs1801282 *PPARG* polymorphism had a significantly higher BMI and waist-to-hip ratio compared to the *CC* genotype [20]. PPARG is found mainly in white adipose tissue and is involved in the final differentiation of the pre-adipocyte into a mature adipocyte [21]. The hypothesis that PPARG may be involved in bone metabolism stems from the fact that osteoblasts and adipocytes share a precursor cell [21,22]. Although an exact conclusion has yet to be reached, there is much research into the role of *PPARG* in osteoblast differentiation and activity. The results obtained by Akune et al. in the study of *PPARG* activation in osteoblastogenesis in *PPARG*-deficient heterozygous mice (*Pparg*+/−) showed a 50% reduction in *PPARG* expression. *PPARG* deficiency increased bone mass by stimulating osteoblastogenesis from bone marrow progenitor cells, which confirms the negative role of *PPARG* activation in bone formation [23]. In order to be able to draw precise conclusions, these results must be confirmed in well-controlled randomized trials in humans. Mbalaviele et al. showed that activation of the PPARG pathway by the endogenous PPARG ligand blocks the action of the OPG ligand and thus inhibits the formation and activity of osteoclasts in human mesenchymal stem cells by inhibiting the NF-κB pathway [24]. OPG is a circulating receptor without a transmembrane domain and inhibits osteoclast differentiation by acting as a decoy receptor for RANK, which is expressed on the surface of an osteoclast precursor and interferes with the interaction of RANK with RANKL [25,26,27]. Given that the NF-κB pathway is involved in the cytokine-inhibitory mechanism of adipocyte differentiation by down-regulating *PPARG* expression and the aforementioned PPARG agonist mechanism that inhibits osteoclast differentiation by inhibiting NF-κB transcription, we can assume that the inhibition of osteoclastogenesis by OPG may be directly related to the activation of PPARG [28]. The most common *PPARG* variant is a proline to alanine substitution (Pro12Ala) of the *PPARG2* gene (rs1801282). In a study by Rhee et al., the mean serum OPG level was significantly lower and the serum total ALP level was significantly higher in women with the Pro12Ala genotype compared to the Pro12Pro genotype, even after adjusting for other confounders. In the haplotype analysis with the 161*C>T* variant for *PPARG*, women with the Ala and *T* alleles showed significantly lower serum OPG levels, suggesting a negative impact of these variants on serum OPG levels. These results indicate that the Ala and *T* alleles have a significant impact on the bone resorption. However, this conclusion was not confirmed by the correlation between other studied clinical parameters [29], whereas the involvement of PPARG in the regulation of systemic energy metabolism and the regulation of osteocyte function in mice were confirmed. Deletion of *PPARG* under the Dmp1-Cre promoter-driver caused mice to have an increased bone mass and high energy metabolism. It was concluded that PPARG places a molecular brake on the metabolic activity in osteocytes. As they age or enter pathological conditions, osteocytes produce SASP proteins that lead to unbalanced remodeling in bone tissue, loss of bone mass, and fractures. Osteocytes have become targets for selective PPARG modulators being tested, which are expected to affect bone mass by regulating sclerostin levels [30].

A statistically significant difference was observed for the rs213045 (*ECE1*) variant. The *T* allele was more common in women with osteopenia compared to the controls (*p* = 0.037). The *GG* and *GT* of *ECE1* rs213045 variants occurred statistically more often in the control group than in women with osteopenia, with 57.1% vs. 50.5% and with 37.4% vs. 35.8%, while the *TT* genotype occurred more often in women with osteopenia compared to the control group, with 13.8% vs. 5.5% (*p* = 0.037). With the *GG* genotype as a reference, the codominant and recessive crude models were associated with an increased risk of osteopenia (*p* < 0.05). 

In both polymorphic variants of the *ECE1* gene, minor alleles were associated with a lower birth weight of the study participants: for allele *T* vs. *G* (rs213045) vs. *G* (*p* = 0.011), and for *C* vs. *A* (*p* = 0.038). The results of a comparison of the haplotype frequencies of *ECE1* gene variants showed that the haplotype containing two major *GA* alleles (rs213045, rs213046) reduces the risk of osteopenia in our population (*p* = 0.032). The only statistically significant difference observed for densitometric results was lower median Z-score values for the *T* allele compared to the *G* allele for the rs213045 variant of the *ECE1* gene (*p* = 0.021).

In their study, Liu et al. analyzed the effect of the *ECE1* gene polymorphism on osteoporosis in 281 postmenopausal women. They showed that it is related to the incidence of osteoporosis, through effects on estrogen concentrations or interactions between estrogen and nitric oxide (NO). They showed that the *GT* genotype 894*G>T* (rs213045) affects plasma testosterone and osteocalcin concentrations, and the *TT* genotype is related to BMD. However, different from our study, bone mineral density was significantly lower in women with the *GT* genotype compared to the *TT* genotype, despite the fact that patients with the *GT* genotype had higher plasma levels of testosterone and osteocalcin. Increased levels of testosterone and osteocalcin should reflect the activation of osteoblasts. This indicates that the relationship between the 894*G>T* polymorphism may be complex and related to the influences of various factors [31]. However, the genetic variant rs213045 *ECE1* may be related to the risk of osteoporosis. Moreover, in our project, we studied more than twice as many female patients. 

Also, Hansen et al. looked for an association of the occurrence of single-nucleotide polymorphisms (SNPs) of *ECE1* (*G>T*) rs213045 and *ECE1* (*A>C)* rs213046 with osteoporosis in postmenopausal women. In a study involving 3548 postmenopausal Caucasian women, they showed that women with the *CC* genotype had statistically fewer fractures over the age of 50 compared to women with the *AA* and *AC* genotypes of the rs213046 polymorphism (*p* < 0.001). They also showed that the *AC* genotype was associated with osteoporosis. However, they found no differences in BMD values when comparing the *GG*, *GT*, and *TT* genotypes of the rs213045 polymorphism [10]. A summary of studies for ECE1 and PPARG polymorphisms in various populations, along with our study, is presented in Table 6.

In a study carried out on mice, Johnson et al. showed that the variability in the genomic region containing the *ECE1* allele influences bone size and accounts for 40% of the variability in bone biomechanics and BMD in the cross of recombinant HcB-8 and HcB-23 congenic strains. According to their research, *ECE1*-dependent END-1 signaling affects bone physiology in in vitro, ex vivo, and in vivo studies. The addition of exogenous END-1 to immortalized murine osteoblast cultures increases mineralization and reduces sclerostin production by regulating miR1263. It was also observed that the addition of END-1 produced effects on bone tissue similar to those seen with mechanical loading [10,32,33].

## 5. Conclusions

This study found that women with osteoporosis had statistically significantly lower body mass and BMI values compared to the control group. Additionally, our results suggest that the haplotype containing two major *GA* alleles of *ECE1* (rs213045, rs213046) may reduce the risk of osteopenia, as seen in our population. In the densitometric results, lower median Z-score values were observed for the *T* allele compared to the *G* allele for the rs213045 variant of the *ECE1* gene. Moreover, the *T* allele of rs213045 was more common in women with osteopenia compared to the controls. The analysis of clinical and densitometric parameters depending on the allele distribution indicated a statistically significantly higher BMI in carriers of the *G* allele of the *PPARG* rs1801282 variant. In summary, our study suggests that the *ECE1* rs213045 variant may increase the risk of osteopenia. A limitation of our study is the relatively small and non-homogeneous groups of women studied. Despite having 606 individuals, this number may not have been sufficient to identify associations for low-frequency variants. Because our study has numerous limitations, the conclusions obtained require further confirmation and point to further research directions. However, any research in this field has great potential, because understanding the mechanisms that may influence the development of osteoporosis may allow for effective therapies in the future. Early diagnosis of the disease, even before the onset of symptoms, and the use of appropriate drugs adapted to the patient’s genotype may slow or inhibit its further development. 

## Figures and Tables

**Table 1:** Comparison of clinical and densitometric data of women with osteoporosis and osteopenia and the control group.

Variables	Controls (*N* = 254)	Osteoporosis (*N* = 245)	Osteopenia (*N* = 109)	*p*
Age (years)	54 ± 8 [32; 73]	57 ± 9 [34; 74]	53 ± 8 [30; 72]	0.007
Birth weight (g)	3600 [3445; 3790]	3000 [2870; 3300]	3200 [2965; 3435]	0.001
First menstruation (years)	14 [12; 15]	13 [11; 15]	13 [11; 15]	0.526
Last menstruation (years)	50 [47; 53.5]	49 [45; 51]	50 [47; 52]	0.037
Years of reproduction	36.38 ± 5.35	35.59 ± 5.05	36.20 ± 4.93	0.713
Years after menopause	6.5 [3; 9]	10.5 [6; 15]	6 [3; 9.5]	<0.001
Pregnancy number	2 [1; 2]	2 [1; 3]	2 [1; 2]	0.941
Weight (kg)	68.81 ± 12.14	61.21 ± 9.14	65.42 ± 11.15	<0.001
Height (cm)	162.66 ± 5.69	160.21 ± 5.15	162.84 ± 5.05	0.001
BMI (kg/m^2^)	26.04 ± 4.55	23.80 ± 3.09	24.67 ± 3.97	0.003
L2-L4 BMD (g/cm^2^)	1.18 [1.12; 1.25]	0.83 [0.77; 0.87]	0.97 [0.93; 1.03]	<0.001
L2-L4 YA (%)	98 [94; 105]	69 [65; 73]	81 [77.5; 86]	<0.001
L2-L4 AM (%)	108 [101.5; 114.5]	78 [74; 82]	89 [84; 94]	<0.001
LS T-score	−0.17 [−0.67; 0.57]	−3.06 [−3.48; −2.71]	−1.88 [−2.18; −1.40]	<0.001
LS Z-score	0.56 [−0.10; 1.33]	−1.62 [−2.09; −1.17]	−0.88 [−1.34; −0.41]	<0.001

Mean ± SD, median (interquartile range [IQR]), p ANOVA, or Kruskal–Wallis test.

**Table 2:** Minor allele frequencies (MAFs) and results of the Hardy–Weinberg equilibrium test for the studied SNPs.

SNP	Alleles	MAF in Europe *	Controls		Osteoporosis		Osteopenia	
			MAF	HWE *p*	MAF	HWE *p*	MAF	HWE *p*
rs1801282	*C>G*	0.1203	0.173	1.000	0.173	0.823	0.378	0.114
rs213045	*G>T*	0.2873	0.242	0.865	0.247	0.170	0.317	0.077
rs213046	*A>C*	0.0954	0.071	1.000	0.071	1.000	0.101	0.596

* 1000 genomes (Europe).

**Table 3:** The allele frequencies of rs1801282 (*PPARG*), rs213045, and rs213046 (*ECE1*) variants and bone mass reduction susceptibility.

SNP	Allele	Controls (*N* = 508)	Osteoporosis (*N* = 490)	OR (95% CI)	Chi^2^ *p*	Osteopenia (*N* = 218)	OR (95% CI)	Chi^2^ *p*
rs1801282	*C*	420 (0.827)	405 (0.827)	1.00 (0.72–1.39)	χ^2^ = 0.0001 0.991	188 (0.862)	0.76 (0.49–1.19)	χ^2^ = 1.421 0.233
	*G*	88 (0.173)	85 (0.173)			30 (0.138)		
rs213045	*G*	385 (0.758)	369 (0.753)	1.03 (0.77–1.37)	χ^2^ = 0.031 0.859	149 (0.683)	1.45 (1.02–2.06)	χ^2^ = 4.339 0.037
	*T*	123 (0.242)	121 (0.247)			69 (0.317)		
rs213046	*A*	472 (0.929)	455 (0.929)	1.01 (0.62–1.63)	χ^2^ = 0.001 0.972	196 (0.899)	1.47 (0.84–2.57)	χ^2^ = 1.874 0.171
	*C*	36 (0.071)	35 (0.071)			22 (0.101)		

Pearson’s p.

**Table 4:** Logistic regression analysis of the associations between bone mass reduction susceptibility and rs1801282, rs213045, and rs213046 in different genetic models.

SNP	Genotypes	Controls (*N* = 254)	Osteoporosis (*N* = 245)	OR (95%CI)	*p*	*p* Adj.	Osteopenia (*N* = 109)	OR (95%CI)	*p*	*p* Adj.
rs1801282	*CC*	173 (68.1)	168 (68.6)	1.00	0.927	0.836	83 (76.1)	1.00	0.190	0.296
	*CG*	74 (29.1)	69 (28.2)	0.96 (0.65–1.42)			22 (20.2)	0.62 (0.36–1.07)		
	*GG*	7 (2.8)	8 (3.3)	1.18 (0.42–3.32)			4 (3.7)	1.19 (0.34–4.18)		
	Dominant	81 (31.9)	77 (31.4)	0.98 (0.67–1.43)	0.912	0.664	26 (23.9)	0.67 (0.40–1.12)	0.119	0.174
	Recessive	247 (97.2)	237 (96.7)	1.19 (0.43–3.34)	0.739	0.615	105 (96.3)	1.34 (0.39–4.69)	0.647	0.761
	Overdominant	180 (70.9)	176 (71.8)	0.95 (0.65–1.41)	0.811	0.796	87 (79.8)	0.62 (0.36–1.06)	0.071	0.120
rs213045	*GG*	145 (57.1)	143 (58.3)	1.00	0.491	0.324	55 (50.5)	1.00	0.037	0.325
	*GT*	95 (37.4)	83 (33.9)	0.89 (0.61–1.29)			39 (35.8)	1.08 (0.67–1.76)		
	*TT*	14 (5.5)	19 (7.8)	1.38 (0.66–2.85)			15 (13.8)	2.82 (1.28–6.23)		
	Dominant	109 (42.9)	102 (41.6)	0.95 (0.67–1.35)	0.772	0.247	54 (49.5)	1.31 (0.83–2.05)	0.245	0.136
	Recessive	240 (94.5)	226 (92.2)	1.44 (0.71–2.94)	0.313	0.695	94 (86.2)	2.74 (1.27–5.89)	0.011	0.615
	Overdominant	159 (62.6)	162 (66.1)	0.86 (0.59–1.24)	0.411	0.134	70 (64.2)	0.93 (0.58–1.49)	0.769	0.216
rs213046	*AA*	219 (86.2)	211 (86.1)	1.00	0.999	0.512	87 (79.8)	1.00	0.182	0.209
	*AC*	34 (13.4)	33 (13.5)	1.01 (0.60–1.69)			22 (20.2)	1.63 (0.90–2.94)		
	*CC*	1 (0.4)	1 (0.4)	1.04 (0.06–16.70)			0 (0.0)	—		
	Dominant	35 (13.8)	34 (13.9)	1.01 (0.61–1.68)	0.975	0.558	22 (20.2)	1.58 (0.88–2.85)	0.131	0.510
	Recessive	253 (99.6)	244 (99.6)	1.04 (0.06–16.67)	0.980	0.279	109 (100.0)	—	1.000	0.128
	Overdominant	220 (86.6)	212 (86.5)	1.01 (0.60–1.69)	0.978	0.718	220 (86.6)	1.64 (0.91–2.95)	0.107	0.339

*p*-value adjusted by age and BM.

**Table 5:** The association between the haplotypes of *ECE1* genes rs213045 and rs213046 and the bone mineral density susceptibility.

ECE1 Haplotypes	Frequency				*p* Controls vs. Osteopenia	*p* Controls vs. Osteoporosis
	All	Osteopenia	Osteoporosis	Controls		
GA	0.741	0.678	0.753	0.756	0.032	0.918
TA	0.183	0.221	0.176	0.174	0.138	0.925
TC	0.075	0.096	0.071	0.069	0.202	0.874

Chi-square *p*-value.

**Table 6:** Summary of studies in various populations for ECE1 and PPARG polymorphisms.

Publication	No.	Population	Gene	Conclusions and Results	Reference
Hansen et al.	3564	Postmenopausal women and predominantly non-Hispanic caucasian	*ECE1* rs213045 *ECE1* rs213046	The CC (rs213046) genotype was associated with fewer fractures, whereas the AC genotype was associated with osteoporosis.	[10]
Reza-Lopez et al.	314	Mexican adults with T2D	*PPARG* rs1801282	The G rs1801282 allele is associated with a higher BMI and waist circumference than in CC homozygotes.	[19]
Li et al.	70,137	Meta-analysis	*PPARG* rs1801282	The carriers of the G allele of the rs1801282 *PPARG* polymorphism had a significantly higher BMI and waist-to-hip ratio compared to the CC genotype.	[20]
Rhee et al.	239	Healthy Korean women	*PPARG*	In the haplotype analysis with the 161C>T variant for *PPARG*, women with the Ala and T alleles showed significantly lower serum OPG levels.	[29]
Liu et al.	281	Postmenopausal women of Chinese Han nationality	*ECE1* rs213045	The GT genotype rs213045 affects plasma testosterone and osteocalcin concentrations, and the TT genotype is related to BMD.	[31]
Uzar et al.	608	Postmenopausal woman	*ECE1* rs213045, rs213046, *PPARG* 1801282	The women with osteoporosis had statistically significantly lower body mass and BMI values compared to the control group. The haplotype containing two major GA alleles of *ECE1* (rs213045, rs213046) may reduce the risk of osteopenia. The analysis of clinical and densitometric parameters depending on the allele distribution indicated a statistically significantly higher BMI in carriers of the G allele of the *PPARG* rs1801282 variant.	This work

T2D—type 2 diabetes, BMI—body mass index, BMD—bone mineral density.

## Data Availability

The original contributions presented in the study are included in the article, further inquiries can be directed to the corresponding author.
